# Temporal trends in cardiac arrest and cancer-related mortality among adults in the United States, 1999–2023

**DOI:** 10.1186/s40959-026-00483-1

**Published:** 2026-04-13

**Authors:** Reja Ahmad, Muhammad Salik Uddin, Urooj Amjad, Hasibullah Aminpoor, Muhammad Ahsan, Aafia Imran, Muhammad Zubair Farooq, Abdur Rafay Bilal, Asim Sajjad, Muhammad Ahmed, Muhammad Mubariz, Saad Ahmed Waqas

**Affiliations:** 1 Ziauddin Medical College, Karachi, Pakistan; 2 Department of Medicine, Dow University of Health Sciences, Karachi, Pakistan; 3 Faculty of Medicine, Kabul University of Medical Sciences “Abu Ali Ibn Sina”, Ata Turk Avenue, Jamal Mena, 3rd District, Kabul, 1001 Afghanistan; 4 Internal Medicine, Doctors Hospital at Renaissance, Edinburg, TX USA

**Keywords:** Cancer, Cardiac arrest, Cardio-oncology, Mortality

## Abstract

**Background:**

Cardiac arrest (CA) remains a major contributor to cardiovascular-related mortality in the United States. The coexistence of cancer significantly exacerbates overall disease burden. This study investigates CA and cancer-related trends and demographic disparities in adults from 1999 to 2023.

**Methods:**

This retrospective analysis of CDC WONDER data investigates the trends in mortality associated with CA in patients with cancer. Using Joinpoint regression analysis, the study calculated age-adjusted mortality rates (AAMR) per 100,000 individuals and corresponding annual percentage changes (APC), along with 95% confidence intervals.

**Results:**

Between 1999 and 2023, CA and cancer–related mortality accounted for 1,503,315 deaths. With an AAPC of -2.3 (95% CI: -2.4 to -2.1, *p* < 0.001), the overall AAMR decreased from 37.3 in 1999 to 21.1 in 2023. Adult men had higher AAMRs than women (men: 48.4; women: 30.4) in 1999 to (men: 25.7; women: 17.8) in 2023, with decline for both sexes [men: AAPC: -2.5, *p* < 0.001; women: AAPC: -2.2, *p* < 0.001]. AAMRs varied significantly by race, for NH Black individuals (57.6 to 28.1), NH American Indians (22.1 to 15.5), Hispanics (44.6 to 24.7) and NH Whites (34.4 to 19.5) from 1999 to 2023 respectively. The greatest decline in AAMR was observed in middle-aged adults (AAPC: -2.3, *p* < 0.001). Regionally, the highest decline was seen in South region (AAPC: -3.3, *p* < 0.001). AAMRs varied by state, from 5.2 in West Virginia to 55.3 in California during 2023.

**Conclusion:**

This study reveals significant demographic and geographic disparities in CA and cancer-related mortality in U.S. adults from 1999 to 2023, with a disproportionately high burden observed among older adults, males, and NH Black individuals. These findings underscore the urgent need for targeted, equity-driven public health strategies for high-risk groups.

**Supplementary Information:**

The online version contains supplementary material available at 10.1186/s40959-026-00483-1.

## Introduction

Cancer and cardiac arrest remain two of the most formidable challenges in modern healthcare, collectively accounting for a significant portion of global mortality and imposing a massive burden on public health. In the United States, out-of-hospital cardiac arrest remains responsible for an estimated 350,000 annually with about a 90% fatality rate [1]. Considering the nation’s aging population and the rising prevalence of cardiovascular disease-projected to affect over 60% of U.S. adults by 2050–the burden of cardiac arrest is expected to increase substantially in the coming decades [2]. Similarly, cancer continues to be a leading cause of death globally, accounting for approximately 10 million fatalities each year [3]. In the United States, cancer imposes a significant financial challenge, with nation’s cancer care expenditures estimated at $208.9 billion in 2020, a figure expected to rise further in the future [4].

The bidirectional relationship between cancer and cardiac arrest represents a critical, evolving area of clinical concern with substantial implications for patient care and survival [5]. Current evidence reveals that individuals with cancer face a significantly elevated risk of sudden cardiac death, with specific malignancies and demographic groups exhibiting higher mortality rates [6, 7]. A range of pathophysiological mechanisms contribute to this relationship, including direct cardiotoxicity from chemotherapy—particularly anthracyclines, tyrosine kinase inhibitors, and immunotherapies—along with radiation-induced heart injury, cancer-associated thrombosis, metabolic disturbances, and overlapping cardiovascular risk factors [8, 9]. These intersections create a difficult clinical balance, as cardiovascular complications have emerged as a leading cause of non-cancer mortality among survivors, while pre-existing heart disease may restrict cancer therapy and worsen overall outcomes [10].

Although prior studies have evaluated cardiac arrest mortality trends in the United States and cancer mortality trends independently, most investigations have examined these conditions separately or explored cardiac arrest and cancer outcomes in relation to other cardiovascular comorbidities such as diabetes or hypertension [11–14]. However, national analyses specifically examining deaths in which cardiac arrest and cancer are concurrently documented on the death certificate remain limited. In addition, many existing studies rely on out-of-hospital cardiac arrest registries or institutional datasets, which may not capture the full national burden. Consequently, long-term national mortality patterns involving the co-occurrence of cardiac arrest and cancer remain insufficiently characterized. Therefore, this study addresses this gap by analyzing CDC WONDER data to evaluate trends of cardiac arrest and cancer–related mortality among adults in U.S. from 1999 to 2023, with comparisons by sex, age, race, and geography to inform public health strategy.

## Methodology

### Study setting and population

Deaths occurring within the United States related to cardiac Arrest and cancer were extracted from the Centers for Disease Control and Prevention’s Wide-Ranging Online Data for Epidemiologic Research (CDC WONDER) database. Detailed query parameters and extraction procedures are provided in Supplementary Table 1 (Reproducibility Appendix). The Multiple Cause-of-Death Public Use record death certificates were analyzed to identify cases where cardiac arrest and cancer both were listed as the multiple cause of death. The definitions of cardiac Arrest and cancer used in this study are consistent with prior publications examining these conditions in the US. The study population included individuals with cardiac arrest and cancer identified using International Classification of Diseases, 10th Revision (ICD-10) codes, including I46 for cardiac arrest and C00-C97 for cancer. Geographic data were analyzed at both the state and regional levels, with all U.S. states and the District of Columbia included. Regions were classified based on the U.S. Census Bureau definitions, dividing the United States into Northeast, Midwest, South, and West. Urban-rural classifications followed the 2013 National Center for Health Statistics Urban-Rural Classification Scheme, categorizing areas into metropolitan (large and fringe metro), medium/small metro, and rural (micropolitan or noncore counties).

### Data abstraction

Cardiac arrest and cancer-related deaths, population sizes, and place of death were extracted from the CDC WONDER database from 1999 to 2023, including deaths occurring in medical facilities (inpatient, outpatient, emergency room, or status unknown), home, hospice, and nursing home/long-term care facilities. Demographic variables such as age, sex, race/ethnicity, geographic location (state and urban-rural classifications), and cause of death rankings were also included. State-level analyses were conducted using 2023 data, the most recent year available in the CDC WONDER dataset. Urban-rural analyses were limited to 1999–2020 due to the limited availability of the urbanization variable in the CDC WONDER database. Age groups were categorized as young adults (25–44 years), middle-aged adults (45–64 years), and old-aged adults (65–85 + years), and sex was classified as male and female. Race and ethnicity classifications followed CDC WONDER standard categories, including NH (Non-Hispanic) American Indian or Alaska Native, NH Black or African American, NH White, and Hispanic or Latino. The rankable cause of death feature in CDC WONDER was utilized to obtain death counts and rates for the top 15 leading causes of death among individuals with these conditions.

### Statistical analysis

Crude and age-adjusted mortality rates (AAMRs) per 100,000 individuals were determined. Crude mortality rates were calculated by dividing the number of cardiac arrest and cancer-related deaths by the corresponding U.S. population for each year. AAMRs were standardized to the 2000 U.S. population to account for differences in age distribution across years. Trend analysis was conducted using log-linear regression models, and Annual Percent Change (APC) was calculated to evaluate significant changes in mortality rates over time. The Joinpoint Regression Program (Joinpoint version 5.2.0, National Cancer Institute) was used to detect inflection points where significant shifts in mortality trends occurred. APCs with 95% confidence intervals (CI) were calculated at the identified join points using the Monte Carlo permutation test, and trends were considered increasing or decreasing if the mortality slope was significantly different from zero using two-tailed t-testing. Statistical significance was set at *p* < 0.05.

## Results

**Fig. 1:**
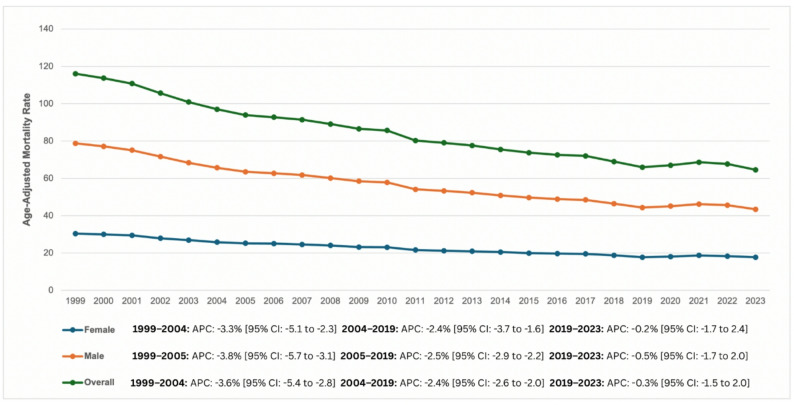
Trends and disparities in cardiac arrest and cancer-related AAMR per 100,000 U.S adults stratified by sex from 1999 to 2023

Between 1999 and 2023, cardiac arrest and cancer-related mortality accounted for 1,503,315 deaths (Table 1). By 2023, the AAMR for cardiac arrest mortality declined from 37.31 (95% CI: 37.02 to 37.59) per 100,000 in 1999 to 21.14 (95% CI: 20.96 to 21.31) in 2023, with an AAPC of -2.28 (95% CI: -2.44 to -2.14; *p* < 0.001). However, the rate of decline varied across the study period, with a significant decline from 1999 to 2004 (APC: -3.56%; 95% CI: -5.41 to -2.79; *p* < 0.001) and from 2004 to 2019 (APC: -2.37%; 95% CI: -2.63 to -2.01; *p* = 0.002), followed by a slower non-significant decline from 2019 to 2023 (APC: -0.34%; 95% CI: -1.51 to 1.96; *p* = 0.644) (Fig. 1, Supplementary Table 2 and Supplementary Table 11).

**Table 1:** Frequency and age-adjusted rates per 100,000 in adults with cardiac arrest and cancer concomitantly, stratified by sex, race, age, and census region

Category	Deaths	Population	AAMR 1999 (95% CI)	AAMR 2023 (95% CI)	AAPC (95% CI)
Overall	1,503,315	5,163,131,262	37.31 (37.02–37.59)	21.14 (20.96–21.31)	–2.3 (–2.4 to − 2.1)
Sex					
Male	800,622	2,491,041,251	48.35 (47.83–48.87)	25.69 (25.40–25.97)	–2.5 (–2.7 to − 2.4)
Female	702,693	2,672,090,011	30.42 (30.08–30.75)	17.76 (17.54–17.97)	–2.2 (–2.4 to − 2.0)
Age					
Young Adults (25–44)	38,830	2,119,405,590	2.57 (2.46–2.68)	1.63 (1.54–1.71)	–1.8 (–2.1 to − 1.5)
Middle-aged Adults (45–64)	353,125	1,942,357,841	23.65 (23.26–24.04)	13.49 (13.25–13.74)	–2.3 (–2.4 to − 2.2)
Old-aged Adults (65–85+)	1,111,360	1,101,367,831	143.27 (142.00–144.53)	80.60 (79.85–81.34)	–2.2 (–2.4 to − 2.1)
Race					
Non-Hispanic White	1,085,519	3,528,912,956	34.38 (34.09–34.68)	19.54 (19.34–19.73)	–2.3 (–2.4 to − 2.2)
Non-Hispanic Black / African American	204,267	602,204,655	57.60 (56.38–58.82)	28.14 (27.51–28.78)	–2.9 (–3.2 to − 2.7)
Non-Hispanic American Indian or Alaska Native	6,389	37,910,158	22.07 (18.43–25.71)	15.52 (13.65–17.39)	–1.2 (–1.9 to − 0.5)
Hispanic or Latino	136,330	702,028,728	44.58 (43.13–46.03)	24.72 (24.13–25.30)	–2.4 (–2.5 to − 2.2)
Census Region					
Northeast	476,867	948,225,258	62.29 (61.48–63.09)	30.94 (30.46–31.43)	–2.8 (–3.2 to − 2.3)
Midwest	156,630	1,110,999,093	17.16 (16.76–17.55)	11.69 (11.41–11.97)	–1.5 (–1.6 to − 1.3)
South	414,506	1,916,380,109	30.97 (30.53–31.41)	13.49 (13.27–13.71)	–3.3 (–3.4 to − 3.0)
West	455,312	1,187,526,802	46.52 (45.81–47.24)	34.77 (34.30–35.23)	–1.2 (–1.4 to − 1.1)

### Sex

Throughout the study period, men consistently exhibited higher AAMRs than women. In 1999, the AAMR for men was 48.35 (95% CI: 47.83 to 48.87) declining to 25.69 (95% CI: 25.40 to 25.97) in 2023 (AAPC of -2.50% [95% CI: -2.66 to -2.36]; *p* < 0.001). Similarly, the AAMR for women decreased from 30.42 (95% CI: 30.08 to 30.75) in 1999 to 17.76 (95% CI: 17.54 to 17.97) in 2023 (AAPC of -2.20% [95% CI: -2.39 to -2.01]; *p* < 0.001) (Fig. 1; Table 1, Supplementary Table 3, Supplementary Table 11).

AAMR trends were broadly similar between males and females across the study period. Among females, mortality declined significantly from 1999 to 2004 (APC: -3.26%; 95% CI: -5.06 to -2.32; *p* = 0.006), followed by a continued significant decline from 2004 to 2019 (APC: -2.38%; 95% CI: -3.70 to -1.62; *p* = 0.005). From 2019 to 2023, the trend stabilized, with a small but non-significant decline observed (APC: -0.16%; 95% CI: -1.71 to 2.40; *p* = 0.894). A comparable pattern was observed among males. Mortality decreased significantly from 1999 to 2005 (APC: -3.80%; 95% CI: -5.66 to -3.14; *p* < 0.001) and continued to decline significantly between 2005 and 2019 (APC: -2.52%; 95% CI: -2.86 to -2.15; *p* < 0.001). In the most recent period, 2019 to 2023, a minor non-significant decline was observed (APC: -0.48%; 95% CI: -1.72 to 2.01; *p* = 0.537).

### Race

**Fig. 2:**
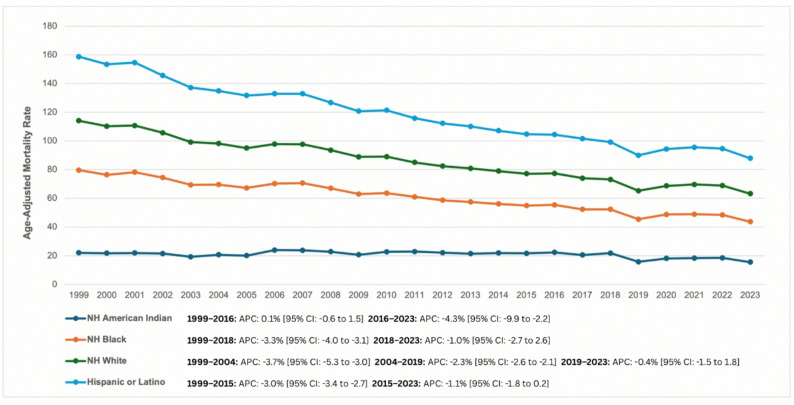
Trends and disparities in cardiac arrest and cancer-related AAMR per 100,000 U.S adults stratified by race from 1999 to 2023

When analyzed by race, the greatest decline in AAMRs was seen among NH Black or African American adults with a mean AAMR of 57.60 (95% CI: 56.38 to 58.82) in 1999 to 28.14 (95% CI: 27.51 to 28.78) in 2023 (AAPC: -2.85 [95% CI: -3.15 to -2.65]; *p* < 0.001), followed by Hispanic or Latino adults, from 44.58 (95% CI: 43.13 to 46.03) to 24.72 (95% CI: 24.13 to 25.30) (AAPC: -2.35 [95% CI: -2.53 to -2.17]; *p* < 0.001), the NH White adults from 34.38 (95% CI: 34.09 to 34.68) to 19.54 (95% CI: 19.34 to 19.73) (AAPC: -2.30 [95% CI: -2.43 to -2.16]; *p* < 0.001), and NH American Indian or Alaska Natives adults from 22.07 (95% CI: 18.43 to 25.71) to 15.52 (95% CI: 13.65 to 17.39) (AAPC: -1.20 [95% CI: -1.91 to -0.55]; *p* < 0.016) (Fig. 2; Table 1, Supplementary Tables 4 and 11).

Among NH Black or African American individuals AAMR declined significantly from 1999 to 2018 (APC: -3.34%; 95% CI: -4.02 to -3.12; *p* = 0.007). From 2018 to 2023, the decline continued at a slower rate but was not statistically significant (APC: -0.96%; 95% CI: -2.71 to 2.61; *p* = 0.348).

Hispanic or Latino individuals experienced a significant decline in AAMR from 1999 to 2015 (APC: -2.96%; 95% CI: -3.38 to -2.67; *p* < 0.001). From 2015 to 2023, the downward trend persisted but was attenuated and no longer statistically significant (APC: -1.12%; 95% CI: -1.78 to 0.23; *p* = 0.076).

NH White individuals demonstrated a significant decline in AAMR from 1999 to 2004 (APC: -3.69%; 95% CI: -5.30 to -3.00; *p* < 0.001), followed by a continued significant decline from 2004 to 2019 (APC: -2.30%; 95% CI: -2.55 to -2.05; *p* < 0.001). Between 2019 and 2023, the trend stabilized with a small non-significant decline (APC: -0.44%; 95% CI: -1.52 to 1.80; *p* = 0.555).

For NH American Indian or Alaska Native individuals, AAMR remained relatively stable from 1999 to 2016 with a small non-significant increase (APC: 0.10%; 95% CI: -0.58 to 1.48; *p* = 0.671). This pattern reversed thereafter, with a significant decline observed from 2016 to 2023 (APC: -4.30%; 95% CI: -9.92 to -2.20; *p* < 0.001).

### Age

AAMRs varied substantially by age group during the study period. Middle-aged adults marked a significant decrease from 23.65 (95% CI: 23.26 to 24.04) in 1999 to 13.49 (95% CI: 13.25 to 13.74) in 2023 (AAPC: -2.26 [95% CI: -2.38 to -2.15]; *p* < 0.001), followed by older adults (65–85+) a decrease from 143.27 (95% CI:142 to 144.53) in 1999 to 80.60 (95% CI: 79.85 to 81.34) in 2023 (AAPC: -2.23 [95% CI: -2.43 to − 2.09]; *p* < 0.001).In contrast, young adults (25–44) had the smallest decrease, from 2.57 (95% CI: 2.46 to 2.68) in 1999 to 1.63 (95% CI: 1.54 to 1.71) in 2023 (AAPC: -1.82 [95% CI: -2.07 to -1.52]; *p* < 0.001) (Table 1, Supplementary Fig. 1, Supplementary Tables 5 and 11).

Young adults (25–44 years) experienced a significant decline in AAMR from 1999 to 2002 (APC: -4.99%; 95% CI: -7.95 to -2.41; *p* = 0.009). This was followed by a continued but non-significant decline from 2002 to 2012 (APC: -2.26%; 95% CI: -3.76 to 1.73; *p* = 0.118). From 2012 to 2023, the downward trend persisted at a slower rate and remained non-significant (APC: -0.53%; 95% CI: -3.88 to 2.27; *p* = 0.454).

Middle-aged adults (45–64 years) showed a significant decline in AAMR from 1999 to 2005 (APC: -4.00%; 95% CI: -4.97 to -3.38; *p* < 0.001), followed by a further significant decline from 2005 to 2023 (APC: -1.68%; 95% CI: -1.82 to -1.53; *p* < 0.001).

Older adults (65–85+) also experienced a significant decline in AAMR from 1999 to 2019 (APC: -2.70%; 95% CI: -2.92 to -2.55; *p* < 0.001). However, from 2019 to 2023, this pattern leveled off, with a small non-significant increase observed (APC: 0.10%; 95% CI: -1.51 to 3.16; *p* = 0.866).

### Census region

While analyzing the geographic regions, the South, from 30.97 (95% CI: 30.53 to 31.41) in 1999 to 13.49 (95% CI: 13.27 to 13.71) in 2023 (AAPC: -3.26 [95% CI: -3.44 to -2.99]; *p* < 0.001), had the steepest decline out of all the regions, followed by the Northeast, from 62.29 (95% CI: 61.48 to 63.09) in 1999 to 30.94 (95% CI: 30.46 to 31.43) in 2023 (AAPC: -2.76 [95% CI: -3.23 to -2.34]; *p* < 0.001), the Midwest, from 17.16 (95% CI: 16.76 to 17.55) to 11.69 (95% CI: 11.41 to 11.97) (AAPC: -1.46 [95% CI: -1.64 to -1.27]; *p* < 0.001), and the West, from 46.52 (95% CI: 45.81 to 47.24) to 34.77 (95% CI: 34.30 to 35.23) in 2023 (AAPC: -1.24 [95% CI: -1.36 to -1.12]; *p* < 0.001) (Table 1, Supplementary Fig. 2, Supplementary Tables 6 and 11).

In the Northeast, AAMR showed a non-significant decline from 1999 to 2007 (APC: -2.40%; 95% CI: -4.27 to 3.08; *p* = 0.070), followed by a significant decline from 2007 to 2019 (APC: -3.79%; 95% CI: -8.03 to -0.70; *p* = 0.036). From 2019 to 2023, the trend stabilized, with a small non-significant decline observed (APC: -0.35%; 95% CI: -3.38 to 5.01; *p* = 0.753).

In the Midwest, AAMR declined significantly from 1999 to 2009 (APC: -4.28%; 95% CI: -5.03 to -3.69; *p* < 0.001). This pattern then reversed, with a significant increase from 2009 to 2023 (APC: 0.61%; 95% CI: 0.24 to 1.05; *p* = 0.0016).

In the South, AAMR declined significantly from 1999 to 2003 (APC: -4.92%; 95% CI: -7.85 to -3.15; *p* < 0.001), followed by a continued significant decline from 2003 to 2023 (APC: -2.92%; 95% CI: -3.15 to -2.03; *p* = 0.0056).

In the West, AAMR declined significantly from 1999 to 2007 (APC: -2.67%; 95% CI: -3.38 to -2.28; *p* = 0.0016). This was followed by a brief non-significant increase from 2007 to 2010 (APC: 0.53%; 95% CI: -0.99 to 1.38; *p* = 0.661). From 2010 to 2019, mortality again declined significantly (APC: -1.54%; 95% CI: -2.73 to -1.24; *p* < 0.001), before shifting to a modest but significant increase during 2019 to 2023 (APC: 1.05%; 95% CI: 0.02 to 2.94; *p* = 0.045).

### Urbanization

Metropolitan areas experienced a steeper decline in the AAMR than non-metropolitan areas. Metropolitan areas declined from 39.27 in 1999 (95% CI: 38.94 to 39.59) to 22.31 (95% CI: 22.11 to 22.5) in 2020 (AAPC: -2.81 [95% CI: -2.94 to -2.63]; *p* < 0.001). In comparison, the non-metropolitan areas declined from 28.84 (95% CI: 28.26 to 29.42) to 19.81 (95% CI: 19.38 to 20.24) (AAPC: -1.85 [95% CI: -1.99 to -1.70]; *p* < 0.001) (Supplementary Fig. 3, Supplementary Table 7).

In metropolitan areas, AAMR declined significantly from 1999 to 2004 (APC: -3.64%; 95% CI: -5.47 to -2.83; *p* < 0.001), followed by a further significant decline from 2004 to 2020 (APC: -2.55%; 95% CI: -2.71 to -1.89; *p* = 0.002).

In non-metropolitan areas, AAMR also declined significantly from 1999 to 2005 (APC: -3.07%; 95% CI: -4.72 to -2.39; *p* < 0.001), with a continued significant decline from 2005 to 2020 (APC: -1.36%; 95% CI: -1.55 to -1.07; *p* = 0.0008).

### State

A significant variation in AAMRs for cardiac arrest and cancer-related mortality was observed across states in 2023, ranging from 55.25 (95% CI: 54.41 to 56.10) in California to 4.05 (95% CI: 3.05 to 5.29) in Maine. The five states with the highest AAMRs were California (AAMR: 55.25), New York (AAMR: 45.02), Nevada (AAMR: 42.05), Mississippi (AAMR: 38.94), and Arkansas (AAMR: 37.94). In contrast, the lowest AAMRs were observed in Maine (AAMR: 4.05), West Virginia (AAMR: 5.17), Virginia (AAMR: 5.57), Delaware (AAMR: 6.36), and the District of Columbia (AAMR: 6.74) (Supplementary Table 8).

### Place of death

Statistics on the location of death were available for 1,507,832 cardiac arrest and cancer–related mortality cases during the study period 1999–2023. Out these, 694,762 (46.08%) occurred in medical facility in-patient settings, 482,466 (32.00%) in decedent’s home, 224,988 (14.92%) in nursing home/long term care, 52,942 (3.51%) in hospice facilities, and 52,674 (3.49%) in other locations (Supplementary Table 9).

### Top underlying causes of death

We analyzed the top 16 leading underlying causes of death in cardiac arrest and cancer–related mortality. Malignant neoplasms emerged as the leading cause of death with 1,243,580 deaths, followed by heart disease with 131,585 deaths and chronic lower respiratory diseases with 20,297 deaths (Supplementary Table 10).

## Discussion

**Fig. 3:**
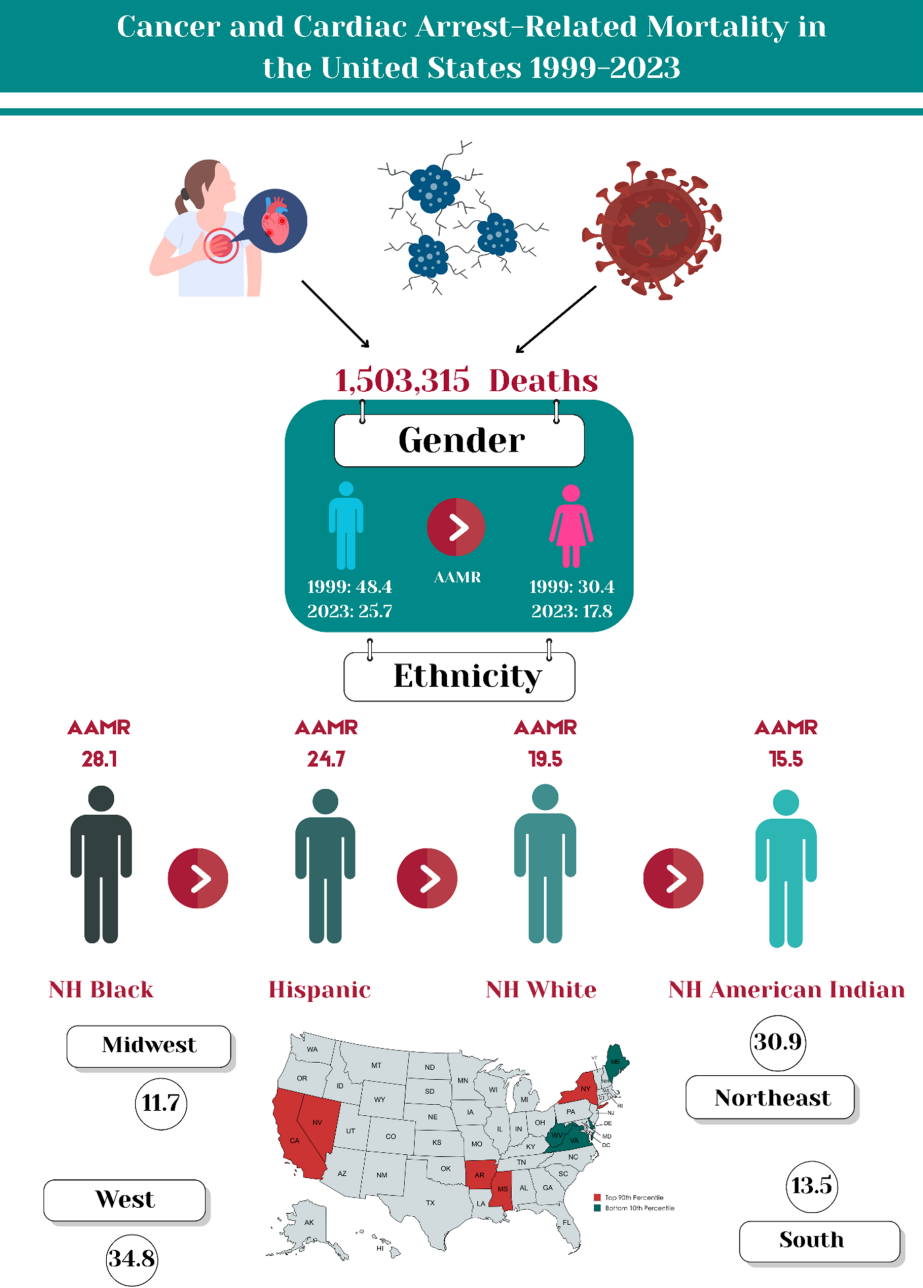
Disparities in cardiac arrest and cancer-related mortality among U.S adults, 1999–2023

This 25-year analysis of cardiac arrest and cancer–related mortality in the United States reveals a story of remarkable progress shadowed by persistent and deeply rooted inequities. While the national death rate declined substantially over the study period, depicting clearly the advances in both oncologic and cardiovascular care, this success was neither uniform nor consistent. The pace of improvement has recently decelerated to a near halt, and the benefits of medical progress have not been shared equally. Stark disparities remain, with men, NH Black individuals, and residents of non-metropolitan areas continuing to experience disproportionately high mortality burdens. Therefore, this discussion will analyze these disparities in detail to conclude why they were faced and continue to persist in an era of significant medical advancement (Fig. 3).

The present study expands upon prior investigations of cardiac arrest and cancer mortality by providing a comprehensive national assessment of deaths in which both conditions are concurrently documented on the death certificate. Using a large national dataset derived from CDC WONDER and spanning a 25-year period, this analysis evaluates long-term temporal trends in cardiac arrest and cancer-related mortality at the population level. In contrast to previous studies that have often focused on cardiac arrest or cancer mortality independently, the present analysis specifically examines the intersection of these conditions while also evaluating disparities across key demographic groups, including sex, race, and age. Additionally, geographic patterns were assessed through urban-rural classification, census region, and state-level analyses, while further characterization of mortality patterns was achieved through evaluation of place of death and underlying causes of death. Together, these multidimensional analyses provide a more comprehensive population-level understanding of the epidemiology and disparities associated with concurrent cardiac arrest and cancer mortality in the United States.

Prior literature has established several mechanisms through which cancer and its treatment may elevate cardiac arrest risk, context that informs but cannot be confirmed by the present mortality data [15, 16]. Cardiotoxic chemotherapies, notably anthracyclines, have been shown to injure cardiomyocytes and create a substrate for lethal arrhythmias, while radiation therapy has been associated with accelerated coronary artery disease and myocardial fibrosis [17, 18]. Cancer itself has also been linked to a chronic inflammatory and hypercoagulable state that may predispose patients to acute thrombotic events capable of precipitating cardiac collapse [19]. Since CDC WONDER death certificates do not capture treatment regimens, comorbidities, or cause-specific pathophysiology, these mechanisms represent plausible contextual explanations for the trends observed rather than findings derived from this study.

The long-term decline in cardiac arrest and cancer–related mortality reflects parallel advancements in both oncology and cardiology. Progress through is consistent with the concurrent rise of the cardio-oncology field and a broader shift toward more targeted therapies with improved cardiovascular safety profiles as documented in the published literature, though the present data cannot isolate these factors as causal drivers [20, 21]. However, the near-stagnation of this trend after 2019 strongly implicates the COVID-19 pandemic. The pandemic created a dual threat: it disrupted cancer care, leading to more advanced disease presentations, while SARS-CoV-2 infection itself directly increased cardiovascular risk in this vulnerable population [22, 23]. This history illustrates two decades of steady progress, followed by a stark demonstration of how quickly a systemic health crisis can halt hard-won public health gains.

The significant gender disparity observed in our cohort reflects a complex interplay of biology, clinical exposures, and behavior that collectively places men at a higher risk while affording women a degree of protection. Biologically, men’s higher mortality is partly explained by their lack of the lifelong cardiovascular protection conferred by estrogen, a key advantage for women that establishes their lower baseline risk for heart disease [24]. This gap may be further widened by differential treatment exposures. Prior research has linked androgen deprivation therapy (ADT) for prostate cancer to increased fatal cardiac events, a mechanism that could contribute to the male excess observed here, though treatment data are not available in WONDER to test this directly [25]. Finally, behavioral patterns compound these risks. Men’s tendency to delay seeking medical care can result in more advanced disease at diagnosis, whereas women’s generally more proactive engagement with the healthcare system often leads to earlier detection and management of both their cancer and comorbid cardiac conditions [26].

Our analysis reveals that a patient’s race remains a powerful predictor of their outcome, with social and economic factors driving profound inequities. NH Black individuals consistently bore the heaviest mortality burden from the dual threat of cancer and cardiac arrest, a disparity that prior literature has attributed to structural inequities and a higher prevalence of cardiovascular comorbidities, though neither factor is directly measurable in this dataset [27, 28]. The Hispanic/Latino population also faced a significant burden, a pattern consistent with published evidence documenting higher rates of metabolic syndrome, a greater incidence of certain cancers, and healthcare barriers that may delay diagnosis in this group, though these factors cannot be verified from death certificate data alone [29, 30]. In contrast, the NH White population’s steady decline reflects broad public health successes; despite a high incidence of cancers like melanoma and breast cancer, the declining mortality in this group is consistent with broader access to cancer screening and advanced treatments documented in the literature, alongside population-level gains from anti-smoking campaigns, though access and treatment variables are not captured in WONDER [31, 32]. Yet, it is the trajectory of the NH American Indian or Alaska Native population that is most alarming. This group showed the least improvement, a finding consistent with prior literature documenting systemic barriers including geographic isolation and limited access to specialized care, though these structural factors are not directly measurable in the present dataset [33, 34]. Mortality patterns stratified by age reveal a paradoxical finding. While the high mortality rates in older adults are anticipated, the most concerning trend may be the one observed in the youngest cohort. The elevated mortality in the elderly is consistent with established clinical knowledge, reflecting the confluence of comorbidities and immunosenescence that attenuates the efficacy of modern therapies [35, 36]. Steady progress was also observed in this group, and particularly among middle-aged adults, who may represent an optimal population for successful cardio-oncology interventions [37]. Particularly notable is the attenuated progress among young adults. Although their absolute mortality rate is low, it has declined more slowly than that of any other age group. This attenuated improvement is consistent with published trends showing rising rates of cardiovascular risk factors such as obesity and diabetes among young adults, alongside increasing diagnoses of aggressive cancers in this demographic, though neither comorbidity burden nor cancer type is ascertainable from WONDER death certificates [38, 39]. Therefore, the data present a complex scenario where, despite significant clinical progress in expected populations, a new and concerning challenge is emerging in a generation that should possess the greatest health advantages.

An examination of geographic mortality patterns compels a focus beyond simple regional differences and toward the growing chasm between metropolitan and non-metropolitan America. In major metropolitan centers, prior research suggests a concentration of academic institutions and NCI-designated cancer centers that may improve access to specialist expertise, clinical trials, and advanced therapies [40, 41]. The lower mortality rates in metropolitan areas observed in this study are consistent with this hypothesis, though the dataset does not capture facility access, treatment type, or individual care pathways. In non-metropolitan areas, prior literature describes a pattern of eroding local healthcare infrastructure, including hospital closures and specialist shortages, that may translate into delays in time-sensitive treatment for both acute cardiac events and progressing cancers [42, 43]. The higher mortality rates in non-metropolitan areas observed in this study are consistent with this structural context, as well as with published evidence of greater socioeconomic distress and higher baseline risk factor prevalence in these communities, though none of these variables are captured in WONDER [44]. This fundamental urban-rural cleavage, as described in prior literature, offers a plausible contextual explanation for the regional patterns observed, with the more urbanized Northeast showing faster gains compared to the more rural Midwest and West, though regional urbanization and resource variables are not directly measurable in this dataset [45]. State-level variation, including elevated rates in states such as Mississippi and New York, is consistent with this broader pattern of structural and demographic heterogeneity documented in the literature, though state-level determinants cannot be isolated from the present data [46]. These observed geographic patterns suggest that place of residence may be a meaningful correlate of mortality risk, a relationship warranting further investigation with data capable of capturing individual-level access, treatment, and socioeconomic factors.

The setting in which these deaths occurred further illuminates the disparities discussed throughout this analysis. While a plurality of deaths happened in an inpatient medical facility, reflecting the acute nature of cardiac arrest, a strikingly large proportion of patients died at home. This distribution is likely not uniform and is instead shaped by the same inequities already identified. Patients in non-metropolitan areas, for example, face geographic barriers that increase the likelihood of dying at home simply because they cannot reach a hospital in time [47]. Similarly, prior research suggests that a patient’s access to inpatient palliative care versus dying at home or in a nursing home may reflect socioeconomic and racial barriers, though individual-level socioeconomic data are not available in WONDER to confirm this relationship in the present cohort [48]. Therefore, the place of death is not just a final statistic but is itself another marker of systemic inequity.

These findings demand targeted action. Based on the disparities identified in this surveillance analysis and consistent with prior literature, cardiovascular risk stratification should be considered a standard component of oncology care, with heightened vigilance for high-risk groups including men, non-Hispanic Black individuals, and young adults with premature risk factors. Policymakers must address the urban-rural divide by investing in the healthcare infrastructure of non-metropolitan areas, funding rural hospitals, and expanding telehealth to bring specialist expertise to remote populations [49, 50]. Finally, researchers must pivot from documenting disparities to designing interventions. Future studies should focus on the unique vulnerabilities of young cancer patients and on developing culturally competent programs to close the mortality gaps in racial and ethnic minorities, especially the American Indian and Alaska Native communities where progress has stalled [51]. Acting on these recommendations is essential to ensure that a patient’s survival is determined by medical science, not by their zip code or race.

### Limitations

This study’s findings are subject to several limitations inherent to death certificate data. Our analysis is contingent on the accurate coding of cancer and cardiac arrest, which may be subject to misclassification. The database also lacks granular clinical data, and therefore does not capture treatment regimens, cancer stage, comorbidities, or socioeconomic variables. As a result, the mechanistic and structural explanations discussed, including the potential roles of cardiotoxic therapies, access to NCI-designated cancer centers, and population-level obesity trends, should be interpreted as contextual interpretations grounded in prior literature rather than direct findings of this analysis. Furthermore, as a population-level analysis, these findings are subject to the ecological fallacy and cannot be used to infer individual risk. Despite these constraints, this study provides a valuable surveillance overview, successfully identifying major national trends and disparities that warrant targeted public health attention.

## Conclusion

In conclusion, this study documents a significant and prolonged decline in cardiac arrest and cancer–related mortality, a clear testament to decades of advancement in cardio-oncology. However, a disproportionately high burden remains among older adults, males, and NH Black individuals. These results should serve as a mandate to shift the national focus from solely developing better treatments to the far more complex challenge of their just and equitable delivery. The next chapter of progress against this disease must be defined by an unwavering commitment to health equity, ensuring that the life-saving gains of the past quarter-century can reach every patient in every community.

## Supplementary Information

Supplementary Material 1.

## Data Availability

The data supporting the findings of this study were obtained from the CDC WONDER online database (Centers for Disease Control and Prevention Wide-ranging Online Data for Epidemiologic Research). The datasets used and analyzed during the current study are publicly available and can be accessed at [CDC WONDER] ( https://wonder.cdc.gov).
