# Supplementary material for: Temporal trends in cardiac arrest and cancer-related mortality among adults in the United States, 1999–2023
Source: Cardiooncology. 2026 Apr 13;12:62. doi: 10.1186/s40959-026-00483-1 (PMC13191940; doi:10.1186/s40959-026-00483-1)
Supplement: Supplementary file 1 — Supplementary Material 1. [file 40959_2026_483_MOESM1_ESM.docx]

**
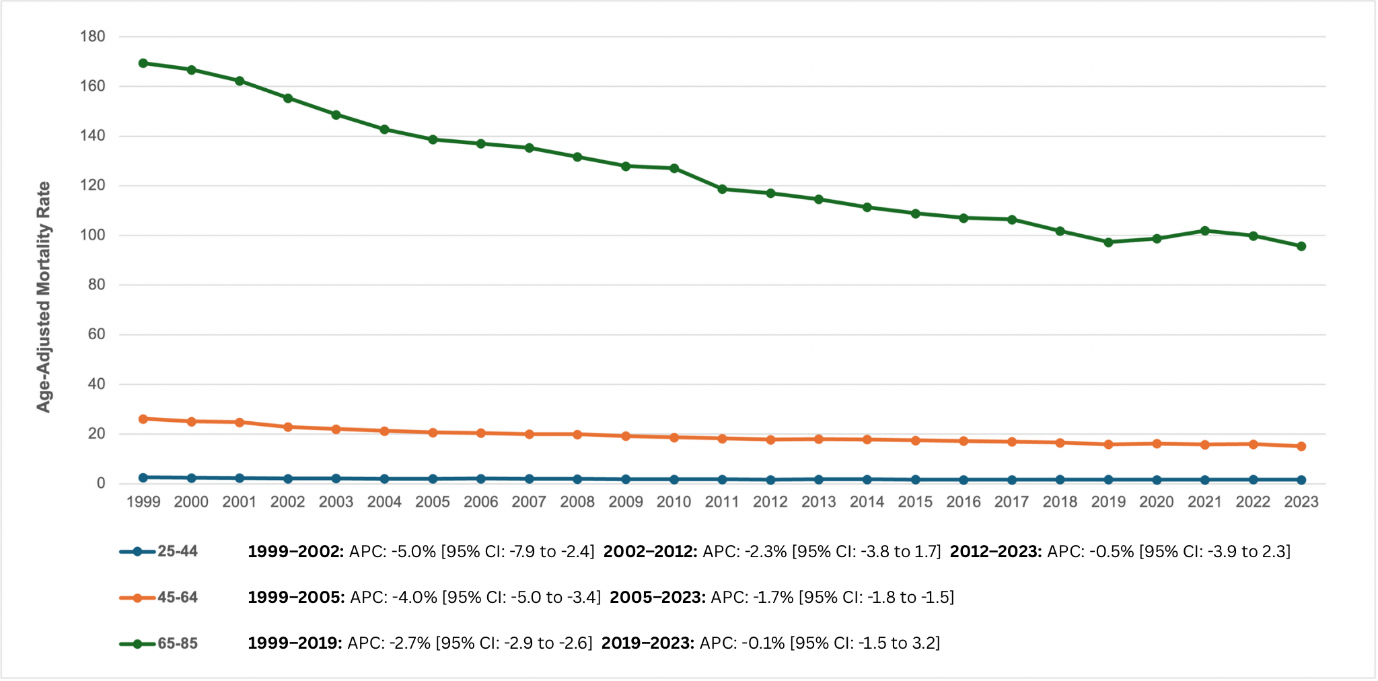
**

**Supplementary Figure 1:** Trends and disparities in cardiac arrest and cancer-related AAMR per 100,000 U.S adults stratified by age from 1999 to 2023.

**
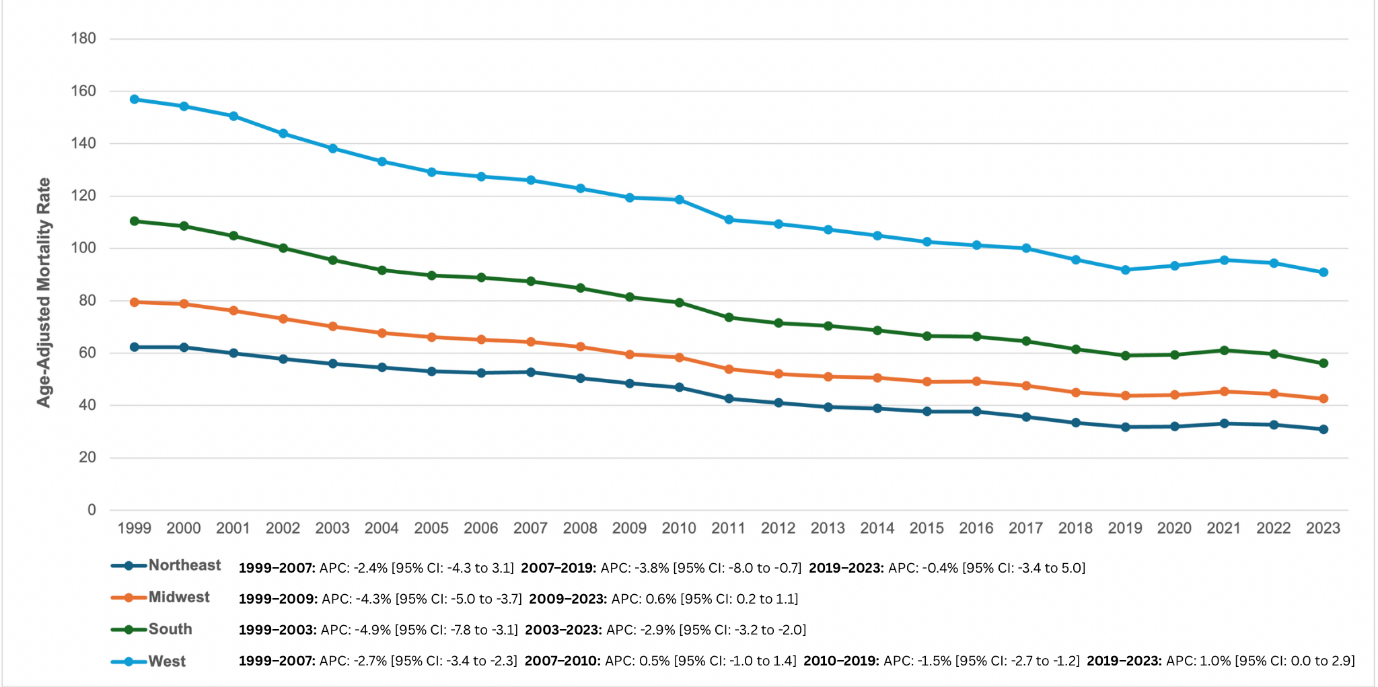
**

**Supplementary Figure 2:** Trends and disparities in cardiac arrest and cancer-related AAMR per 100,000 U.S adults stratified by census region from 1999 to 2023.

**
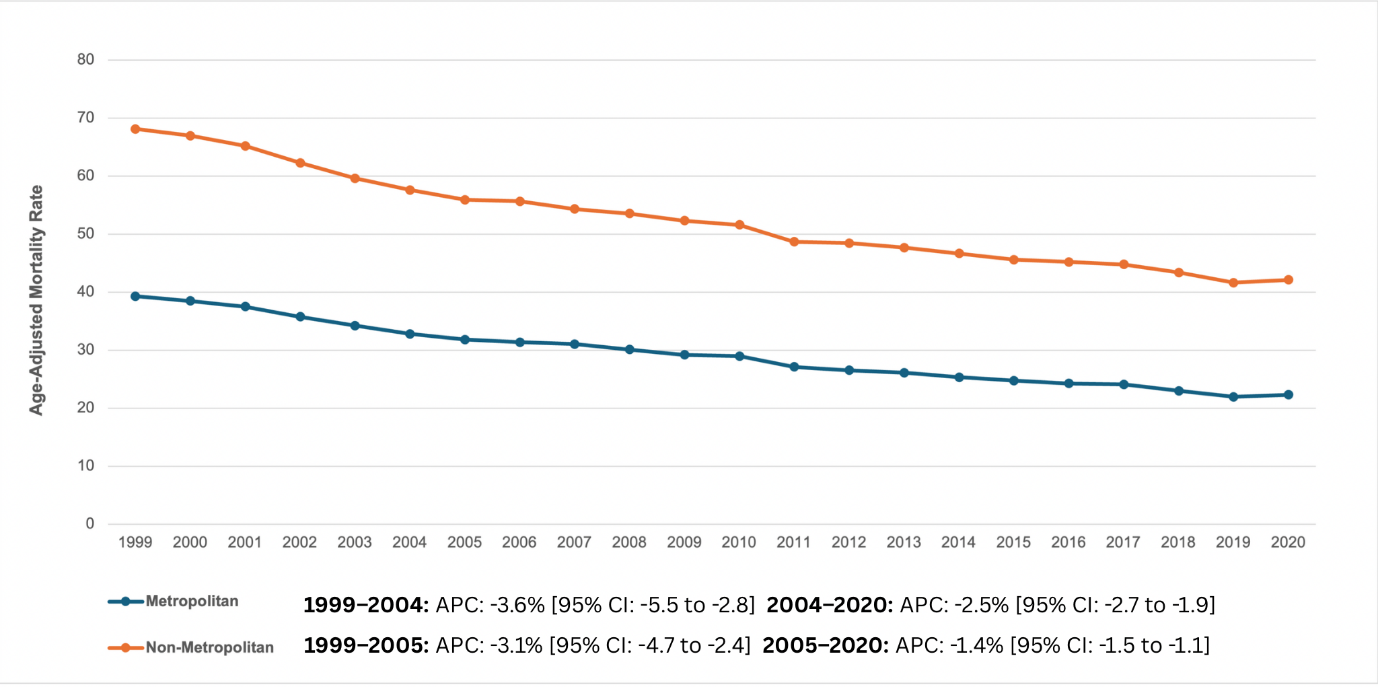
**

**Supplementary Figure 3:** Trends and disparities in cardiac arrest and cancer-related AAMR per 100,000 U.S adults stratified by urbanization from 1999 to 2023.

| Component | Specification |
| --- | --- |
| Data Source | Centers for Disease Control and Prevention (CDC) Wide-ranging Online Data for Epidemiologic Research (CDC WONDER), Multiple Cause of Death Database (1999-2023) derived from the National Vital Statistics System (NVSS) death certificate records. |
| Geographic Coverage | United States – all 50 states and the District of Columbia. |
| Study Period | 1999-2023. |
| Population Included | Adults aged 25-85+ years. |
| Case Definition | Deaths were included if the death certificate listed both cardiac arrest (ICD-10 code I46) and malignant neoplasms (ICD-10 codes C00-C97) anywhere on the death certificate. |
| ICD-10 Codes Used | Cardiac arrest: I46 Malignant neoplasms: C00-C97 |
| Cause-of-Death Fields Used | Codes were identified in multiple cause-of-death fields. Records were included if both I46 and any malignant neoplasm code (C00-C97) were present on the death certificate. |
| Output Measures | Age-adjusted mortality rates (AAMR) |
| Standard Population for Age Adjustment | 2000 U.S. Standard Population using direct standardization. |
| Rate Units | Rates expressed per 100,000 population. |
| Stratified Analyses | Subgroup analyses were performed by sex, race/ethnicity, census region, and state using the grouping variables extracted in the initial query. |

**Supplementary Table 1:** Reproducibility appendix for CDC WONDER data extraction, including database selection, ICD-10 code definitions, filters, and query parameters.

| Year | Overall | Female | Male | Hispanic or Latino | NH American Indian or Alaska Native | NH Black or African American | NH White | Population |
| --- | --- | --- | --- | --- | --- | --- | --- | --- |
| 1999 | 65,998 | 31,393 | 34,605 | 4,043 | 160 | 8,787 | 50,754 | 180,408,769 |
| 2000 | 65,489 | 31,356 | 34,133 | 4,118 | 176 | 8,443 | 50,369 | 181,984,640 |
| 2001 | 64,922 | 31,163 | 33,759 | 4,492 | 185 | 8,882 | 48,901 | 184,305,128 |
| 2002 | 62,963 | 29,975 | 32,988 | 4,231 | 183 | 8,520 | 47,671 | 186,208,028 |
| 2003 | 61,311 | 29,316 | 31,995 | 4,294 | 172 | 8,242 | 46,112 | 188,090,429 |
| 2004 | 59,899 | 28,453 | 31,446 | 4,326 | 201 | 8,229 | 44,714 | 190,205,384 |
| 2005 | 59,288 | 28,296 | 30,992 | 4,563 | 196 | 8,163 | 43,911 | 192,551,384 |
| 2006 | 59,731 | 28,540 | 31,191 | 4,592 | 226 | 8,204 | 44,294 | 195,019,359 |
| 2007 | 60,050 | 28,529 | 31,521 | 4,870 | 231 | 8,507 | 43,963 | 197,403,777 |
| 2008 | 59,823 | 28,312 | 31,511 | 4,850 | 241 | 8,312 | 43,857 | 199,795,090 |
| 2009 | 59,291 | 27,811 | 31,480 | 4,905 | 241 | 8,157 | 43,384 | 202,107,016 |
| 2010 | 59,724 | 28,113 | 31,611 | 5,173 | 250 | 8,057 | 43,335 | 203,891,983 |
| 2011 | 57,542 | 26,925 | 30,617 | 5,252 | 260 | 7,817 | 41,513 | 206,592,936 |
| 2012 | 58,191 | 27,091 | 31,100 | 5,398 | 273 | 7,758 | 42,100 | 208,826,037 |
| 2013 | 58,532 | 27,147 | 31,385 | 5,577 | 279 | 7,944 | 42,065 | 211,085,314 |
| 2014 | 58,425 | 27,201 | 31,224 | 5,724 | 302 | 7,794 | 41,795 | 213,809,280 |
| 2015 | 58,452 | 26,926 | 31,526 | 5,929 | 312 | 7,834 | 41,377 | 216,553,817 |
| 2016 | 58,752 | 27,136 | 31,616 | 6,036 | 331 | 8,022 | 41,429 | 218,641,417 |
| 2017 | 59,745 | 27,482 | 32,263 | 6,464 | 324 | 7,987 | 41,817 | 221,447,331 |
| 2018 | 58,571 | 26,898 | 31,673 | 6,419 | 363 | 7,886 | 40,753 | 223,311,190 |
| 2019 | 57,182 | 25,953 | 31,229 | 6,353 | 267 | 7,904 | 39,595 | 224,981,167 |
| 2020 | 59,223 | 26,791 | 32,432 | 6,892 | 330 | 8,383 | 40,186 | 226,635,013 |
| 2021 | 59,865 | 27,210 | 32,655 | 7,087 | 298 | 8,192 | 40,641 | 228,238,412 |
| 2022 | 61,139 | 27,657 | 33,482 | 7,371 | 313 | 8,272 | 41,267 | 229,508,599 |
| 2023 | 59,207 | 27,019 | 32,188 | 7,371 | 275 | 7,971 | 39,716 | 231,529,762 |

**Supplementary Table 2.** Overall and sex-stratified cardiac arrest and cancer-related mortality among United States adults from 1999 to 2023.

| Year | Overall | Female | Male |
| --- | --- | --- | --- |
| 1999 | 37.31 (37.02–37.59) | 30.42 (30.08–30.75) | 48.35 (47.83–48.87) |
| 2000 | 36.59 (36.31–36.87) | 30.01 (29.68–30.34) | 47.10 (46.59–47.61) |
| 2001 | 35.67 (35.40–35.95) | 29.45 (29.12–29.78) | 45.65 (45.16–46.15) |
| 2002 | 34.00 (33.73–34.26) | 27.91 (27.59–28.23) | 43.76 (43.28–44.24) |
| 2003 | 32.58 (32.32–32.83) | 26.91 (26.61–27.22) | 41.43 (40.97–41.90) |
| 2004 | 31.30 (31.05–31.55) | 25.79 (25.49–26.09) | 39.90 (39.45–40.35) |
| 2005 | 30.40 (30.16–30.65) | 25.23 (24.93–25.52) | 38.32 (37.88–38.75) |
| 2006 | 30.06 (29.81–30.30) | 25.07 (24.77–25.36) | 37.65 (37.23–38.08) |
| 2007 | 29.65 (29.41–29.89) | 24.62 (24.33–24.91) | 37.17 (36.76–37.59) |
| 2008 | 28.91 (28.68–29.14) | 24.05 (23.77–24.33) | 36.12 (35.72–36.53) |
| 2009 | 28.08 (27.86–28.31) | 23.20 (22.92–23.47) | 35.25 (34.86–35.65) |
| 2010 | 27.82 (27.60–28.05) | 23.06 (22.79–23.33) | 34.78 (34.39–35.17) |
| 2011 | 26.11 (25.89–26.32) | 21.65 (21.39–21.91) | 32.48 (32.11–32.85) |
| 2012 | 25.71 (25.50–25.92) | 21.25 (21.00–21.51) | 32.07 (31.70–32.43) |
| 2013 | 25.28 (25.07–25.49) | 20.95 (20.70–21.20) | 31.36 (31.01–31.71) |
| 2014 | 24.63 (24.43–24.84) | 20.53 (20.28–20.78) | 30.32 (29.98–30.67) |
| 2015 | 24.07 (23.87–24.26) | 19.91 (19.67–20.15) | 29.79 (29.45–30.13) |
| 2016 | 23.68 (23.49–23.88) | 19.73 (19.49–19.97) | 29.16 (28.83–29.49) |
| 2017 | 23.51 (23.32–23.70) | 19.52 (19.28–19.76) | 28.96 (28.64–29.28) |
| 2018 | 22.55 (22.36–22.73) | 18.77 (18.55–19.00) | 27.66 (27.35–27.97) |
| 2019 | 21.57 (21.39–21.75) | 17.75 (17.53–17.97) | 26.63 (26.33–26.93) |
| 2020 | 21.90 (21.72–22.08) | 18.05 (17.83–18.27) | 27.05 (26.75–27.35) |
| 2021 | 22.45 (22.27–22.64) | 18.70 (18.48–18.93) | 27.48 (27.17–27.78) |
| 2022 | 22.10 (21.93–22.28) | 18.32 (18.10–18.54) | 27.28 (26.98–27.58) |
| 2023 | 21.14 (20.96–21.31) | 17.76 (17.54–17.97) | 25.69 (25.40–25.97) |

**Supplementary Table 3.** Overall and sex-stratified cardiac arrest and cancer-related AAMR per 100,000 in the United States from 1999-2023.

| Year | Hispanic or Latino (95% CI) | NH American Indian or Alaska Native (95% CI) | NH Black or African American (95% CI) | NH White (95% CI) |
| --- | --- | --- | --- | --- |
| 1999 | 44.58 (43.13–46.03) | 22.07 (18.43–25.71) | 57.60 (56.38–58.82) | 34.38 (34.09–34.68) |
| 2000 | 43.08 (41.69–44.47) | 21.77 (18.38–25.16) | 54.65 (53.46–55.83) | 33.83 (33.53–34.13) |
| 2001 | 43.90 (42.55–45.25) | 21.87 (18.52–25.21) | 56.34 (55.15–57.53) | 32.50 (32.21–32.79) |
| 2002 | 39.86 (38.60–41.13) | 21.52 (18.20–24.84) | 52.90 (51.76–54.04) | 31.27 (30.99–31.55) |
| 2003 | 37.98 (36.78–39.18) | 19.28 (16.21–22.34) | 50.03 (48.93–51.13) | 29.89 (29.61–30.16) |
| 2004 | 36.63 (35.48–37.78) | 20.71 (17.65–23.77) | 48.85 (47.77–49.92) | 28.66 (28.39–28.92) |
| 2005 | 36.64 (35.53–37.76) | 20.10 (17.09–23.11) | 47.15 (46.11–48.20) | 27.77 (27.51–28.03) |
| 2006 | 35.04 (33.97–36.10) | 23.96 (20.62–27.30) | 46.29 (45.27–47.32) | 27.58 (27.32–27.84) |
| 2007 | 35.20 (34.16–36.24) | 23.80 (20.53–27.07) | 46.83 (45.81–47.85) | 26.99 (26.74–27.24) |
| 2008 | 33.19 (32.21–34.17) | 22.78 (19.70–25.86) | 44.25 (43.27–45.22) | 26.51 (26.26–26.76) |
| 2009 | 31.87 (30.93–32.80) | 20.66 (17.85–23.47) | 42.37 (41.43–43.32) | 25.87 (25.63–26.12) |
| 2010 | 32.35 (31.43–33.27) | 22.72 (19.69–25.75) | 40.84 (39.92–41.75) | 25.46 (25.22–25.70) |
| 2011 | 30.80 (29.93–31.67) | 22.90 (19.95–25.85) | 38.14 (37.27–39.02) | 23.96 (23.73–24.20) |
| 2012 | 29.84 (29.01–30.67) | 22.12 (19.33–24.92) | 36.57 (35.73–37.41) | 23.76 (23.53–23.99) |
| 2013 | 29.24 (28.44–30.04) | 21.41 (18.76–24.06) | 36.06 (35.24–36.88) | 23.38 (23.16–23.61) |
| 2014 | 28.13 (27.37–28.89) | 21.92 (19.32–24.53) | 34.17 (33.39–34.95) | 22.88 (22.66–23.11) |
| 2015 | 27.54 (26.81–28.27) | 21.70 (19.18–24.23) | 33.23 (32.48–33.99) | 22.23 (22.02–22.45) |
| 2016 | 27.04 (26.33–27.75) | 22.36 (19.84–24.88) | 33.06 (32.31–33.81) | 21.94 (21.72–22.15) |
| 2017 | 27.51 (26.81–28.20) | 20.50 (18.17–22.83) | 31.82 (31.10–32.54) | 21.73 (21.52–21.95) |
| 2018 | 26.02 (25.36–26.68) | 21.82 (19.49–24.14) | 30.47 (29.78–31.16) | 20.83 (20.62–21.04) |
| 2019 | 24.64 (24.02–25.27) | 15.73 (13.78–17.69) | 29.66 (28.99–30.34) | 19.88 (19.68–20.08) |
| 2020 | 25.66 (25.04–26.29) | 18.13 (16.11–20.16) | 30.60 (29.93–31.27) | 19.95 (19.75–20.15) |
| 2021 | 25.83 (25.20–26.45) | 18.30 (16.15–20.45) | 30.60 (29.91–31.28) | 20.77 (20.56–20.98) |
| 2022 | 25.79 (25.18–26.40) | 18.45 (16.36–20.54) | 30.03 (29.37–30.70) | 20.38 (20.18–20.59) |
| 2023 | 24.72 (24.13–25.30) | 15.52 (13.65–17.39) | 28.14 (27.51–28.78) | 19.54 (19.34–19.73) |

**Supplementary Table 4.** Cardiac arrest and cancer-related AAMR per 100,000 stratified by race in the United States from 1999-2023.

| Year | Young Adults (25-44) | Middle-Aged Adults (45-64) | Old Adults (65-85+) |
| --- | --- | --- | --- |
| 1999 | 2.57 (2.46–2.68) | 23.65 (23.26–24.04) | 143.27 (142.00–144.53) |
| 2000 | 2.42 (2.31–2.52) | 22.68 (22.30–23.05) | 141.67 (140.42–142.92) |
| 2001 | 2.36 (2.26–2.47) | 22.44 (22.07–22.81) | 137.51 (136.29–138.73) |
| 2002 | 2.11 (2.01–2.21) | 20.78 (20.43–21.12) | 132.48 (131.28–133.67) |
| 2003 | 2.20 (2.10–2.30) | 19.87 (19.54–20.20) | 126.57 (125.41–127.73) |
| 2004 | 2.05 (1.95–2.14) | 19.23 (18.91–19.55) | 121.53 (120.40–122.66) |
| 2005 | 2.05 (1.96–2.15) | 18.64 (18.33–18.95) | 117.97 (116.86–119.08) |
| 2006 | 2.10 (2.00–2.20) | 18.36 (18.06–18.66) | 116.58 (115.48–117.67) |
| 2007 | 2.00 (1.90–2.10) | 18.04 (17.74–18.33) | 115.29 (114.22–116.37) |
| 2008 | 1.94 (1.84–2.03) | 17.96 (17.67–18.26) | 111.79 (110.74–112.84) |
| 2009 | 1.89 (1.79–1.99) | 17.37 (17.09–17.65) | 108.70 (107.67–109.73) |
| 2010 | 1.78 (1.69–1.88) | 16.95 (16.67–17.23) | 108.36 (107.34–109.38) |
| 2011 | 1.78 (1.69–1.88) | 16.46 (16.19–16.73) | 100.47 (99.50–101.44) |
| 2012 | 1.67 (1.58–1.76) | 16.10 (15.83–16.37) | 99.31 (98.36–100.26) |
| 2013 | 1.78 (1.69–1.88) | 16.28 (16.01–16.55) | 96.53 (95.61–97.46) |
| 2014 | 1.78 (1.69–1.87) | 16.12 (15.85–16.38) | 93.51 (92.61–94.41) |
| 2015 | 1.73 (1.63–1.82) | 15.74 (15.48–16.00) | 91.40 (90.53–92.28) |
| 2016 | 1.68 (1.59–1.77) | 15.58 (15.32–15.84) | 89.84 (88.98–90.70) |
| 2017 | 1.67 (1.58–1.76) | 15.26 (15.01–15.52) | 89.51 (88.67–90.36) |
| 2018 | 1.72 (1.63–1.81) | 14.82 (14.57–15.07) | 85.27 (84.46–86.08) |
| 2019 | 1.73 (1.64–1.82) | 14.15 (13.90–14.39) | 81.44 (80.66–82.22) |
| 2020 | 1.67 (1.58–1.76) | 14.55 (14.30–14.80) | 82.54 (81.76–83.31) |
| 2021 | 1.63 (1.54–1.71) | 14.19 (13.94–14.43) | 86.12 (85.32–86.93) |
| 2022 | 1.73 (1.64–1.82) | 14.27 (14.02–14.52) | 83.95 (83.18–84.72) |
| 2023 | 1.63 (1.54–1.71) | 13.49 (13.25–13.74) | 80.60 (79.85–81.34) |

**Supplementary Table 5.** Cardiac arrest and cancer-related AAMR per 100,000 stratified by age in the United States from 1999-2023.

| Year | Northeast | Midwest | South | West |
| --- | --- | --- | --- | --- |
| 1999 | 62.29 (61.48 – 63.09) | 17.16 (16.76 – 17.55) | 30.97 (30.53 – 31.41) | 46.52 (45.81 – 47.24) |
| 2000 | 62.19 (61.39 – 62.99) | 16.64 (16.25 – 17.03) | 29.74 (29.32 – 30.17) | 45.72 (45.02 – 46.42) |
| 2001 | 59.99 (59.21 – 60.77) | 16.27 (15.89 – 16.65) | 28.59 (28.17 – 29.00) | 45.71 (45.02 – 46.40) |
| 2002 | 57.74 (56.98 – 58.50) | 15.41 (15.04 – 15.78) | 26.97 (26.57 – 27.37) | 43.77 (43.10 – 44.44) |
| 2003 | 55.97 (55.22 – 56.72) | 14.22 (13.87 – 14.58) | 25.33 (24.94 – 25.71) | 42.67 (42.02 – 43.32) |
| 2004 | 54.56 (53.82 – 55.29) | 13.12 (12.78 – 13.46) | 24.02 (23.65 – 24.39) | 41.45 (40.81 – 42.08) |
| 2005 | 53.07 (52.35 – 53.79) | 13.02 (12.68 – 13.35) | 23.54 (23.18 – 23.90) | 39.51 (38.89 – 40.12) |
| 2006 | 52.47 (51.76 – 53.19) | 12.71 (12.39 – 13.04) | 23.68 (23.32 – 24.03) | 38.57 (37.97 – 39.17) |
| 2007 | 52.77 (52.06 – 53.48) | 11.55 (11.24 – 11.86) | 23.12 (22.77 – 23.47) | 38.65 (38.06 – 39.25) |
| 2008 | 50.45 (49.76 – 51.14) | 11.98 (11.66 – 12.29) | 22.42 (22.08 – 22.76) | 38.04 (37.46 – 38.62) |
| 2009 | 48.42 (47.74 – 49.09) | 11.11 (10.81 – 11.41) | 21.85 (21.52 – 22.19) | 38.01 (37.43 – 38.58) |
| 2010 | 46.96 (46.30 – 47.63) | 11.37 (11.07 – 11.68) | 21.02 (20.70 – 21.34) | 39.25 (38.67 – 39.83) |
| 2011 | 42.64 (42.02 – 43.27) | 11.25 (10.95 – 11.55) | 19.78 (19.47 – 20.09) | 37.35 (36.79 – 37.91) |
| 2012 | 41.02 (40.41 – 41.63) | 11.11 (10.81 – 11.40) | 19.37 (19.07 – 19.68) | 37.81 (37.26 – 38.36) |
| 2013 | 39.39 (38.80 – 39.99) | 11.66 (11.36 – 11.95) | 19.39 (19.09 – 19.69) | 36.71 (36.18 – 37.25) |
| 2014 | 38.88 (38.29 – 39.46) | 11.69 (11.39 – 11.99) | 18.09 (17.80 – 18.37) | 36.20 (35.68 – 36.73) |
| 2015 | 37.73 (37.16 – 38.30) | 11.36 (11.07 – 11.65) | 17.45 (17.17 – 17.72) | 35.99 (35.47 – 36.50) |
| 2016 | 37.72 (37.15 – 38.29) | 11.49 (11.19 – 11.78) | 17.08 (16.81 – 17.35) | 34.95 (34.44 – 35.45) |
| 2017 | 35.68 (35.14 – 36.23) | 11.92 (11.63 – 12.22) | 17.00 (16.73 – 17.26) | 35.51 (35.01 – 36.01) |
| 2018 | 33.40 (32.87 – 33.93) | 11.63 (11.34 – 11.91) | 16.47 (16.21 – 16.73) | 34.20 (33.71 – 34.68) |
| 2019 | 31.75 (31.24 – 32.25) | 12.02 (11.73 – 12.31) | 15.32 (15.08 – 15.57) | 32.69 (32.22 – 33.15) |
| 2020 | 32.00 (31.49 – 32.51) | 12.03 (11.74 – 12.32) | 15.32 (15.07 – 15.56) | 34.07 (33.60 – 34.54) |
| 2021 | 33.11 (32.60 – 33.63) | 12.26 (11.97 – 12.56) | 15.67 (15.42 – 15.92) | 34.52 (34.04 – 35.00) |
| 2022 | 32.67 (32.17 – 33.18) | 11.85 (11.57 – 12.14) | 15.09 (14.85 – 15.33) | 34.80 (34.33 – 35.27) |
| 2023 | 30.94 (30.46 – 31.43) | 11.69 (11.41 – 11.97) | 13.49 (13.27 – 13.71) | 34.77 (34.30 – 35.23) |

**Supplementary Table 6.** Cardiac arrest and cancer-related AAMR per 100,000 stratified by census region in the United States from 1999-2023.

| Year | Metropolitan | Non-Metropolitan |
| --- | --- | --- |
| 1999 | 39.27 (38.94 – 39.59) | 28.84 (28.26 – 29.42) |
| 2000 | 38.47 (38.15 – 38.79) | 28.46 (27.89 – 29.03) |
| 2001 | 37.51 (37.19 – 37.82) | 27.66 (27.10 – 28.23) |
| 2002 | 35.75 (35.45 – 36.05) | 26.50 (25.95 – 27.05) |
| 2003 | 34.21 (33.92 – 34.50) | 25.41 (24.88 – 25.94) |
| 2004 | 32.78 (32.49 – 33.06) | 24.82 (24.29 – 25.34) |
| 2005 | 31.82 (31.55 – 32.10) | 24.08 (23.57 – 24.59) |
| 2006 | 31.35 (31.08 – 31.62) | 24.30 (23.79 – 24.81) |
| 2007 | 31.05 (30.78 – 31.32) | 23.27 (22.77 – 23.76) |
| 2008 | 30.10 (29.84 – 30.36) | 23.43 (22.93 – 23.93) |
| 2009 | 29.18 (28.92 – 29.43) | 23.11 (22.62 – 23.60) |
| 2010 | 28.96 (28.71 – 29.22) | 22.62 (22.14 – 23.11) |
| 2011 | 27.10 (26.85 – 27.34) | 21.57 (21.10 – 22.04) |
| 2012 | 26.53 (26.29 – 26.77) | 21.91 (21.44 – 22.38) |
| 2013 | 26.09 (25.85 – 26.32) | 21.58 (21.12 – 22.04) |
| 2014 | 25.34 (25.11 – 25.56) | 21.30 (20.84 – 21.75) |
| 2015 | 24.75 (24.53 – 24.97) | 20.82 (20.37 – 21.27) |
| 2016 | 24.26 (24.05 – 24.48) | 20.94 (20.49 – 21.39) |
| 2017 | 24.09 (23.88 – 24.31) | 20.69 (20.25 – 21.13) |
| 2018 | 22.99 (22.78 – 23.19) | 20.37 (19.93 – 20.81) |
| 2019 | 21.94 (21.74 – 22.14) | 19.67 (19.24 – 20.09) |
| 2020 | 22.31 (22.11 – 22.50) | 19.81 (19.38 – 20.24) |

**Supplementary Table 7.** Cardiac arrest and cancer-related AAMR per 100,000 stratified by urban-rural class in the United States from 1999-2020.

| State | Age-Adjusted Rate (95% CI) |
| --- | --- |
| Oklahoma | 11.02 (9.84 – 12.20) |
| Texas | 10.52 (10.07 – 10.97) |
| South Carolina | 10.72 (9.75 – 11.69) |
| Arkansas | 37.94 (35.53 – 40.35) |
| Utah | 11.98 (10.42 – 13.54) |
| Tennessee | 13.14 (12.20 – 14.09) |
| South Dakota | 17.23 (14.22 – 20.23) |
| Ohio | 14.25 (13.50 – 14.99) |
| Colorado | 7.71 (6.87 – 8.55) |
| Nebraska | 20.21 (17.98 – 22.45) |
| Louisiana | 17.04 (15.70 – 18.39) |
| Montana | 13.09 (10.85 – 15.33) |
| Wyoming | 8.13 (5.70 – 11.26) |
| Indiana | 7.77 (7.03 – 8.51) |
| Washington | 13.19 (12.27 – 14.10) |
| California | 55.25 (54.41 – 56.10) |
| Alabama | 20.05 (18.70 – 21.39) |
| North Dakota | 20.41 (16.80 – 24.02) |
| Alaska | 11.90 (9.01 – 15.42) |
| Georgia | 24.51 (23.42 – 25.60) |
| North Carolina | 7.28 (6.72 – 7.85) |
| Kentucky | 11.67 (10.56 – 12.77) |
| Missouri | 10.76 (9.86 – 11.65) |
| Idaho | 12.62 (10.83 – 14.41) |
| Kansas | 18.11 (16.39 – 19.83) |
| Michigan | 8.16 (7.56 – 8.76) |
| Oregon | 13.18 (12.00 – 14.36) |
| Iowa | 19.59 (17.93 – 21.25) |
| Nevada | 42.05 (39.50 – 44.61) |
| New Mexico | 12.27 (10.67 – 13.87) |
| West Virginia | 5.17 (4.10 – 6.43) |
| Vermont | 12.46 (9.85 – 15.56) |
| Mississippi | 38.94 (36.42 – 41.46) |
| Delaware | 6.36 (4.84 – 8.21) |
| Arizona | 16.67 (15.68 – 17.67) |
| Virginia | 5.57 (5.01 – 6.13) |
| Pennsylvania | 17.88 (17.11 – 18.64) |
| Wisconsin | 9.81 (8.95 – 10.67) |
| Illinois | 11.13 (10.48 – 11.77) |
| Minnesota | 8.88 (8.04 – 9.73) |
| Maine | 4.05 (3.05 – 5.29) |
| New Jersey | 34.63 (33.32 – 35.94) |
| New Hampshire | 17.58 (15.28 – 19.89) |
| Rhode Island | 13.53 (11.22 – 15.83) |
| Connecticut | 13.55 (12.29 – 14.81) |
| Hawaii | 17.48 (15.25 – 19.72) |
| Maryland | 7.30 (6.55 – 8.05) |
| District of Columbia | 6.74 (4.55 – 9.63) |
| Florida | 13.80 (13.31 – 14.30) |
| New York | 45.02 (44.00 – 46.03) |
| Massachusetts | 35.07 (33.58 – 36.57) |

**Supplementary Table 8.** Cardiac arrest and cancer-related AAMR per 100,000 stratified by state classification in the United States in 2023.

| Year | Medical Facility | Nursing Home/Long Term Care | Hospice Facility | Decedent's Home | Other |
| --- | --- | --- | --- | --- | --- |
| 1999 | 33,279 | 11,016 | 0 | 19,048 | 2,130 |
| 2000 | 32,945 | 11,042 | 0 | 18,948 | 2,338 |
| 2001 | 32,595 | 11,195 | 0 | 18,654 | 2,472 |
| 2002 | 31,112 | 10,804 | 0 | 18,489 | 2,554 |
| 2003 | 29,482 | 10,535 | 682 | 18,304 | 2,208 |
| 2004 | 28,837 | 9,968 | 960 | 17,861 | 2,279 |
| 2005 | 28,538 | 10,190 | 1,525 | 17,110 | 1,910 |
| 2006 | 28,411 | 9,952 | 1,897 | 17,671 | 2,000 |
| 2007 | 28,113 | 9,500 | 2,156 | 18,026 | 2,255 |
| 2008 | 27,496 | 9,161 | 2,382 | 17,071 | 1,713 |
| 2009 | 26,557 | 8,956 | 2,356 | 17,318 | 1,710 |
| 2010 | 27,582 | 9,547 | 2,718 | 19,171 | 1,883 |
| 2011 | 26,254 | 9,119 | 2,715 | 18,372 | 1,669 |
| 2012 | 26,190 | 9,065 | 2,946 | 19,299 | 1,791 |
| 2013 | 26,211 | 8,679 | 2,868 | 19,844 | 2,230 |
| 2014 | 26,062 | 8,779 | 2,712 | 20,067 | 1,905 |
| 2015 | 26,033 | 8,704 | 2,805 | 20,207 | 1,846 |
| 2016 | 26,023 | 8,587 | 2,699 | 20,588 | 1,855 |
| 2017 | 26,187 | 8,517 | 2,826 | 21,109 | 2,118 |
| 2018 | 25,324 | 8,101 | 2,464 | 20,943 | 2,072 |
| 2019 | 25,065 | 7,602 | 2,671 | 20,190 | 2,155 |
| 2020 | 25,484 | 6,313 | 2,247 | 23,755 | 2,324 |
| 2021 | 26,120 | 6,148 | 2,137 | 23,020 | 2,433 |
| 2022 | 27,677 | 6,304 | 2,164 | 22,226 | 2,765 |
| 2023 | 27,185 | 7,204 | 2,012 | 20,744 | 2,059 |

**Supplementary Table 9.** Cardiac arrest and cancer-related mortality among United States adults stratified by place of death from 1999-2023.

| Cause of Death | Total Deaths (1999–2023) |
| --- | --- |
| Malignant neoplasms (C00-C97) | 1,243,580 |
| Diseases of heart (I00-I09,I11,I13,I20-I51) | 131,585 |
| Chronic lower respiratory diseases (J40-J47) | 20,297 |
| Diabetes mellitus (E10-E14) | 12,731 |
| Cerebrovascular diseases (I60-I69) | 9,692 |
| Essential hypertension and hypertensive renal disease (I10,I12,I15) | 9,221 |
| Septicemia (A40-A41) | 7,053 |
| Alzheimer disease (G30) | 3,719 |
| Nephritis, nephrotic syndrome and nephrosis (N00-N07,N17-N19,N25-N27) | 3,746 |
| Chronic liver disease and cirrhosis (K70,K73-K74) | 2,810 |
| Accidents (unintentional injuries) (V01-X59,Y85-Y86) | 2,750 |
| Atherosclerosis (I70) | 2,513 |
| Pneumonitis due to solids and liquids (J69) | 2,564 |
| COVID-19 (U07.1) | 3,156 |
| In situ neoplasms, benign neoplasms and neoplasms of uncertain or unknown behavior (D00-D48) | 1,838 |
| Influenza and pneumonia (J09-J18) | 1,249 |

**Supplementary Table 10.** Cardiac arrest and cancer-related deaths stratified by top 16 underlying causes of death in the United States from 1999-2023.

| Category | Subcategory | Time Interval | APC (95% CI) |
| --- | --- | --- | --- |
| Gender | Female | 1999–2004 | -3.3 (-5.1 to -2.3) |
|  |  | 2004–2019 | -2.4 (-3.7 to -1.6) |
|  |  | 2019–2023 | -0.2 (-1.7 to 2.4) |
|  | Male | 1999–2005 | -3.8 (-5.7 to -3.1) |
|  |  | 2005–2019 | -2.5 (-2.9 to -2.2) |
|  |  | 2019–2023 | -0.5 (-1.7 to 2.0) |
| Race | American Indian or Alaska Native | 1999–2016 | 0.1 (-0.6 to 1.5) |
|  |  | 2016–2023 | -4.3 (-9.9 to -2.2) |
|  | Black or African American | 1999–2018 | -3.3 (-4.0 to -3.1) |
|  |  | 2018–2023 | -1.0 (-2.7 to 2.6) |
|  | White | 1999–2004 | -3.7 (-5.3 to -3.0) |
|  |  | 2004–2019 | -2.3 (-2.6 to -2.1) |
|  |  | 2019–2023 | -0.4 (-1.5 to 1.8) |
|  | Hispanic or Latino | 1999–2015 | -3.0 (-3.4 to -2.7) |
|  |  | 2015–2023 | -1.1 (-1.8 to 0.2) |
| Age | Young Adults | 1999–2002 | -5.0 (-7.9 to -2.4) |
|  |  | 2002–2012 | -2.3 (-3.8 to 1.7) |
|  |  | 2012–2023 | -0.5 (-3.9 to 2.3) |
|  | Middle-Aged Adults | 1999–2005 | -4.0 (-5.0 to -3.4) |
|  |  | 2005–2023 | -1.7 (-1.8 to -1.5) |
|  | Old-Aged Adults | 1999–2019 | -2.7 (-2.9 to -2.6) |
|  |  | 2019–2023 | -0.1 (-1.5 to 3.2) |
| Census Region | Northeast | 1999–2007 | -2.4 (-4.3 to 3.1) |
|  |  | 2007–2019 | -3.8 (-8.0 to -0.7) |
|  |  | 2019–2023 | -0.4 (-3.4 to 5.0) |
|  | Midwest | 1999–2009 | -4.3 (-5.0 to -3.7) |
|  |  | 2009–2023 | 0.6 (0.2 to 1.1) |
|  | South | 1999–2003 | -4.9 (-7.8 to -3.1) |
|  |  | 2003–2023 | -2.9 (-3.2 to -2.0) |
|  | West | 1999–2007 | -2.7 (-3.4 to -2.3) |
|  |  | 2007–2010 | 0.5 (-1.0 to 1.4) |
|  |  | 2010–2019 | -1.5 (-2.7 to -1.2) |
|  |  | 2019–2023 | 1.0 (0.0 to 2.9) |
| Urbanization | Metropolitan | 1999–2004 | -3.6 (-5.5 to -2.8) |
|  |  | 2004–2020 | -2.5 (-2.7 to -1.9) |
|  | Non-Metropolitan | 1999–2005 | -3.1 (-4.7 to -2.4) |
|  |  | 2005–2020 | -1.4 (-1.5 to -1.1) |
| Overall |  | 1999–2004 | -3.6 (-5.4 to -2.8) |
|  |  | 2004–2019 | -2.4 (-2.6 to -2.0) |
|  |  | 2019–2023 | -0.3 (-1.5 to 2.0) |

**Supplementary Table 11.** Summary APCs of cardiac arrest and cancer-related AAMR per 100,000 in the United States from 1999-2023.
